# Saw Palmetto

**Published:** 1898-11

**Authors:** 


Saw Palmetto (Sabal Serrulata, Serenoa Serrulata). Its History, Botany, Chemistry, Pharmacology, Provings, Clinical Experience, and Therapeutic Applications. By Edwin M. Hale, M. D., author of *New Remedies*, *Practice of Medicine*, etc. Philadelphia: Boericke & Tafel, 1898. Price, cloth, 50 cents; by mail, 55 cents.

The Editor of this journal owes the publishers an apology for not noticing this book before. The great number of books awaiting notice is, however, the cause.

Saw Palmetto is a drug that has been before the profession for some years, but only in an empirical way. There have been no authentic provings to entitle it to a place in the materia medica. Dr. Hale has sought to remedy this defect.

The writer has never been satisfied with the work of Dr. Hale in building up the materia medica. It has always seemed superficial. Too much space has been occupied in recording the comments, superficial observations, and chance toxic effects of old-school writers, and too many provings have been made with massive doses, which have prevented any observations of the characteristic actions which homœopathists need in order to prescribe intelligently and successfully. These massive doses produce only gross physiological effects, which are not available in prescribing.

These objections crop out in the manual now under notice. We think it would be well if Dr. Hale would publish a supplement containing provings of dynamic preparations. Then the work would be complete.

